# Computational study of paroxetine-like inhibitors reveals new molecular insight to inhibit GRK2 with selectivity over ROCK1

**DOI:** 10.1038/s41598-019-48949-w

**Published:** 2019-09-10

**Authors:** Seketoulie Keretsu, Swapnil P. Bhujbal, Seung Joo Cho

**Affiliations:** 1 Department of Biomedical Sciences, College of Medicine, Chosun University, Gwangju, 501-759 Republic of Korea; 2 Department of Cellular Molecular Medicine, College of Medicine, Chosun University, Gwangju, 501-759 Republic of Korea

**Keywords:** Computational chemistry, Computational models, Computer modelling, Structure-based drug design

## Abstract

The G-protein coupled receptor kinase 2 (GRK2) regulates the desensitization of beta-adrenergic receptors (β-AR), and its overexpression has been implicated in heart failure. Hence, the inhibition of GRK2 is considered to be an important drug target for the treatment of heart failure. Due to the high sequence similarity of GRK2 with the A, G, and C family (AGC family) of kinases, the inhibition of GRK2 also leads to the inhibition of AGC kinases such as Rho-associated coiled-coil kinase 1 (ROCK1). Therefore, unraveling the mechanisms to selectively inhibit GRK2 poses an important challenge. We have performed molecular docking, three dimensional quantitative structure activity relationship (3D-QSAR), molecular dynamics (MD) simulation, and free energy calculations techniques on a series of 53 paroxetine-like compounds to understand the structural properties desirable for enhancing the inhibitory activity for GRK2 with selectivity over ROCK1. The formation of stable hydrogen bond interactions with the residues Phe202 and Lys220 of GRK2 seems to be important for selective inhibition of GRK2. Electropositive substituents at the piperidine ring and electronegative substituents near the amide linker between the benzene ring and pyrazole ring showed a higher inhibitory preference for GRK2 over ROCK1. This study may be used in designing more potent and selective GRK2 inhibitors for therapeutic intervention of heart failure.

## Introduction

Heart failure is a condition in which the heart fails to produce sufficient myocardial contraction needed to effectively circulate blood throughout the body. The low circulation of blood is mitigated by the release of catecholamines by the sympathetic nervous system^1,2^. Catecholamines bind to the β-adrenergic receptor (β-AR) on the cell surface and activate the downstream release of cAMP, which induces the positive inotropic needed for myocardial contraction of the heart^3^. The stimulated β-AR is desensitized through the phosphorylation of its serine and threonine residues by G protein-coupled receptor kinase 2 (GRK2)^4–6^. The phosphorylation by GRK2 induces arrestin binding at the β-AR, thereby blocking the pathway responsible for increased myocardial contraction^7–9^. Hence, the desensitization of β-AR by inhibition of GRK2 is considered as a potential route for heart failure treatment^10^.

GRK2 is a serine/threonine kinase and is one of the members of A, G, and C family (AGC family) of kinases. AGC kinases play a vital role in cell survival, insulin signalling, regulation of ion transporters and channels, and blood pressure among others and its aberrant activity has been shown to be implicated in several diseases^11,12^. Due to high sequence and structural similarity at the kinase domain among AGC kinases (~33% identity), the inhibition of GRK2 leads to inhibition of other AGC kinases^13^. The Rho-associated coiled-coil containing kinase 1 (ROCK1) is a member of the AGC kinase family and plays crucial role in several vital cellular functions including gene transcription, proliferation, differentiation, apoptosis and oncogenic transformation^14–16^. Rho-associated coiled-coil containing kinase (ROCK2) is also another member of the AGC kinase family. They are known to play an important role in cell migration and invasion, centrosome duplication, cytokinesis, and apoptosis^17^. Several studies have shown that the inhibition of GRK2 leads to the inhibition of ROCK1 and ROCK2. In addition, cross activity between GRK2 and other AGC kinases such as GRK1, GRK3 and protein kinase A (PKA) have been observed and reported^13,18,19^. Therefore, the selective inhibition of GRK2 is considered to be crucial, to avoid unwanted side effects that may result from the inhibition of other AGC kinases.

The catalytic domain of the AGC kinases is highly conserved and consists of a small lobe (N-lobe) and a large lobe (C-lobe). The active site, where most AGC kinase inhibitors bind, is formed at the intersection between the two lobes^19^. The conserved active site consists of the adenine subsite which is adjacent to the hinge moiety, the ribose subsite, the polyphosphate subsite and the hydrophobic subsite as shown in Fig. S1 (Supplementary Material). The hydrophobic subsite is made up of residues from the p-loop, the αC-Helix and the DFG motif^20^. A comparison of the residues at the active site of GRK2, ROCK1 and ROCK2 are shown in Table S1 (Supplementary Material).

Several GRK2 inhibitors have been reported over the last decade. The natural product balanol potently inhibits GRK2 with an IC_50_ of 50 nM (at 3 μM ATP) but lacks the selectivity against protein kinase A (PKA) and protein kinase C (PKC)^21^. Paroxetine, which is an FDA approved serotonine reuptake inhibitor is modestly potent towards GRK2 with an IC_50_ of 1.1 μM and selective against other GRKs^22^. GSK180736A which was originally developed as an ROCK1 inhibitor is a potent inhibitor of GRK2 (IC_50_ = 0.77 µM) and is selective against other GRKs. However GSK180736 exhibited limited bioavailability^18,23^. CMPD101 and CMPD103 developed by Takeda pharmaceuticals showed high activity for GRK2 with selectivity over other AGC kinases but are not bioavailable^24,25^. Bouley *et al*., developed a series of indazole hybrid compounds that showed high potency for GRK2 but these compounds also showed activity for GRK5, ROCK1 and PKA^26^. Recently, Waldschmidt *et al*., have reported a series of paroxetine-like compounds that showed high inhibitory activity for GRK2 and selectivity over other AGC kinases^13,27^. A study of this series of paroxetine-like compounds with the objective to understand the structural factors that drive its potency and selectivity for GRK2 poses an interesting challenge. Therefore, these paroxetine-like compounds were selected for computational study.

In-silico drug design techniques have emerged as powerful methods in assisting drug discovery^28–30^. In this study, we have performed molecular docking, molecular dynamics simulation and molecular mechanics Poisson-Boltzmann surface area (MM/PBSA) free energy calculations on 53 paroxetine-like compounds^13,27^ to gain detailed insight into the binding interactions and binding stability of the inhibitors. Using three dimensional quantitative structure activity relationship (3D-QSAR) studies, CoMFA models were developed for both GRK2 and ROCK1. The contour maps developed from the CoMFA models were analyzed to understand the structural changes favorable for high activity. The contour map results and docking analyses of individual receptors were co-analysed to identify the crucial interactions and structural properties that are important to increase the inhibitory activity for GRK2 and selectivity over ROCK1.

## Methodology

### Dataset

**Figure 1:**
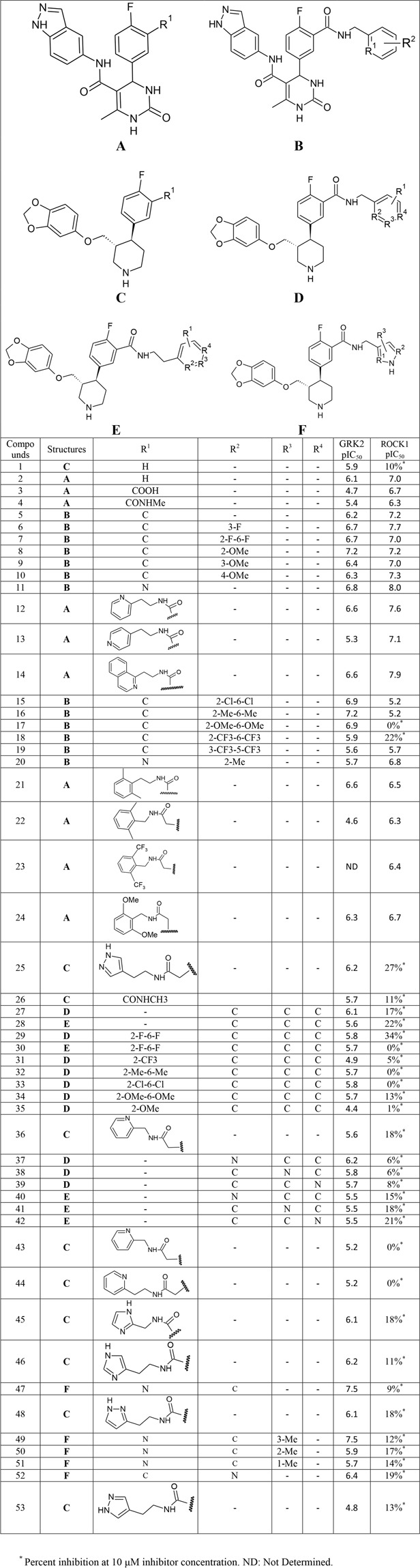
Structure of the paroxetine-like derivatives and their pIC_50_ values for GRK2 and ROCK1.

A series of 53 paroxetine-like compounds having activity values for GRK2 and ROCK1 were collected from recent literature^13,27^. The inhibitory concentration IC_50_ value of the compounds were converted to pIC_50_ (-log IC_50_) values. The series of compounds showed an activity range of 4.42 to 7.52 for GRK2 and an activity range of 5.17 to 7.96 for ROCK1. The structure of the compounds and their pIC_50_ values for GRK2 and ROCK1 are provided in Fig. 1.

The most active compound for GRK2 (compound **47**) showed a pIC_50_ value of 7.523 for GRK2 and showed more than 230-fold selectivity over the other kinases including ROCK1. The most active compound for ROCK1 (compound **11**) showed pIC_50_ value of 6.824 and 7.959 for GRK2 and ROCK1 respectively. The most selective compound (compound **17**) showed activity pIC_50_ value of 6.886 for GRK2 with more than 700-fold selectivity over other kinases including ROCK1.

Based on the binding pose of the most active compound, the rest of the compounds were sketched and minimized using the Tripos force field in Sybyl-X 2.1. The dataset was randomly divided into a test set and a training set. A training set of 33 compounds were used to develop the CoMFA model for GRK2 and 19 compounds were used to validate the model. During the CoMFA model development for ROCK1, 21 compounds were used to build the model after removing all compounds that do not have a specified activity value for ROCK1.

### Protein preparation

The crystal structures of compound **11** with GRK2 (PDB ID: **5HE0**), compound **17** with GRK2 (PDB ID: **5HE2**) and compound **47** with GRK2 (PDB ID: **5UKM**) of the same dataset compounds were retrieved from the protein databank (https://www.rcsb.org/). The crystal structure of ROCK1 (PDB ID: **6E9W**) reported by Hobson *et al*. was used for docking study of the most active compound (compound **11**) with the binding site of ROCK1^31^. The alignment of the amino acid sequences in the kinase domains of GRK2 and ROCK1 are shown in Fig. S2 (Supplementary Material). The missing residues in protein structures were modeled using the homology modeling program MODELLER v9.21^32–34^. The final model after refinement was selected based on statistical potentials (GA341) score and Discrete Optimized Protein Energy (DOPE) score^35^.

### Molecular docking

Docking study of the most active compound (compound **11**) into the binding site of ROCK1 was done using Autodock 4.2.5.1^36^. The crystal structure of ROCK1 in complex with a pyridinylbenzamide based inhibitor (PDB ID: **6E9W**) was already reported in a previous study^31^. The docking protocol was validated by redocking the co-crystalized ligand. The ligand structure was sketched and minimized with the Tripos force field in Sybyl-X 2.1 outside the receptor and then docked to the apo-receptor to perform the redocking. The docked pose showed a root-mean-square deviation (RMSD) value of 1.07 Å.

The docking of the most active compound to ROCK1 was preceded by the preparation of the ligand and the protein. During the protein structure preparation, polar hydrogen atoms were added to the protein. Gasteiger charges were added as partial charges. A grid box of size of 70 × 70 × 70 was created around the ligand to define the area of the receptor to be searched during the docking process. Lamarckian Genetic Algorithm (LGA) was selected to perform the docking. Finally, the docking process was executed to generate 100 docking conformation with 2500000 evaluations per run. The docking results were analyzed using AutoDockTools. Based on its binding energy and important interactions reported in earlier studies, a docked pose was selected and used as input for molecular dynamics simulation studies.

This docking protocol was also used for docking study of compound **17** and compound **47** with ROCK1.

### Molecular dynamics (MD) simulation

MD simulations were carried out in Gromacs 2018^37–41^. The protein topology and structure files were prepared using Amber99SB force field^42^. The ligand topology files were generated with ACYPE package using general AMBER force field (GAFF)^43,44^. The three-point water model (TIP3 water) was used as the solvent. A dodecahedron box was built around the protein-ligand complex and the system was solvated. Sodium ions (NA^+^) were added to the protein-ligand system to neutralize the charge of the system. The system was energy minimized using steepest descent algorithm with the maximum force (F_Max_) set to 1000 KJ/Mol. The system was subjected to constant Number of particles, Volume, and Temperature (NVT) ensemble equilibration for 100 ps to equilibrate the solvent and ions around the protein at 300 K. The temperature coupling was done using modified Berendsen thermostat^45^. Constant number of particle, pressure, and temperature (NPT) ensemble equilibration was performed for 100 ps to stabilize the pressure. During NPT equilibration, Parrinello-Rahman barostat was used for pressure coupling^46^. LINCS algorithm was used to keep the bonds constrained^47^. During NVT and NPT equilibration, the positions of the protein and the ligand were kept restrained. Production MD simulations were carried out for 40 ns without restraints.

### Free energy calculation

Molecular mechanics Poisson–Boltzmann surface area (MM-PBSA) free energy calculation was performed using the g_mmpbsa package^48,49^. The last 5 ns from the production run of the 40 ns MD simulation were used to calculate binding energy. Snapshots were extracted every 50 ps. The binding energy consists of three energetic terms (potential energy in vacuum, polar-solvation energy, and non-polar solvation energy)^50,51^. The vacuum potential energy includes both bonded (angle, bond, and dihedral) and non-bonded (electrostatics and van der Waals) interactions and was calculated based on molecular mechanics force field parameters^42,52^. Polar solvation energy was calculated by solving the Poisson-Boltzmann equation^49,53,54^ and non-polar solvation energy was calculated based on the solvent accessible surface area (SASA) model^55,56^. The binding energy contributed by individual residue was calculated based on the equation given below:

$${\rm{\Delta }}\,{R}_{x}^{BE}=\mathop{\sum }\limits_{i=0}^{n}\,({A}_{i}^{bound}-{A}_{i}^{free})$$

Where, ${\rm{\Delta }}\,{R}_{x}^{BE}$ represents the binding energy of the residue *x*, and ${A}_{i}^{bound}$ and ${A}_{i}^{free}$ are the energy of *i*^th^ atom from *x* residue in bound and unbound forms respectively.

### 3D-QSAR

The comparative molecular field analysis (CoMFA) models were developed for both GRK2 and ROCK1 using Sybyl-X 2.1^57^. In CoMFA model development, the electrostatic field and steric field exerted by the compounds were calculated at each point of a regularly spaced 3D grid around the compounds. A probe atom (sp^3^ carbon of +1 charge and having a van der Waal radius of 1.52 Å) was used to calculate the field exerted. The steric fields were contributed by Lennard-Jones potential and the electrostatic fields were contributed by Coulombic potential.

During the CoMFA model development for GRK2, the binding pose of the most active compound (compound **47**) given in the co-crystal structure (**5UKM**) was used for aligning the dataset compounds. Since the co-crystalized structure of ROCK1 with its most active compound (compound **11**) was not available, the average structure of the most active compound extracted from the last 5 ns of the 40 ns MD simulation was used as a template for developing the CoMFA model for ROCK1.

The dataset compounds were aligned by superimposing on the substructure which was common to all compounds using the ‘database align’ method given in Sybyl-X 2.1. The common substructure used in aligning the dataset compounds was shown in Fig. S3 (Supplementary Material).

**Figure 2:**
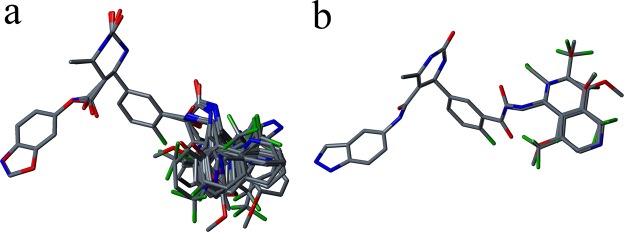
(**a**) Alignment of the dataset compounds used in the CoMFA model development for GRK2. (**b**) Alignment of the dataset compounds used in the CoMFA model development for ROCK1.

The alignments used for developing the CoMFA models for GRK2 and ROCK1 are shown in Fig. 2. Partial least square (PLS) analysis was performed to linearly correlate the 3D-QSAR descriptor values to the activity values. The leave-one-out method was used to derive the cross-validated correlation coefficient (*q*^2^) and optimal number of components (ONC) of the model. The non-cross-validated correlation coefficient (*r*^2^), standard error of estimation and F-test value (F) were evaluated for the CoMFA model based on the ONC value^58^.

### Model validation

The CoMFA models were validated for its robustness and statistical confidence using bootstrapping (BS) analysis. Leave-five-out (LFO) analysis was performed to assess the sensitivity of the models to chance correlation^59^. To test the predictive ability of the models against external test set, predictive correlation coefficient (*r*^2^_*pred*_) was calculated based on the equation given below^60^:

$${{r}^{2}}_{pred}=({\rm{SD}}-{\rm{PRESS}})/{\rm{SD}}$$

where SD represents the squared deviation between the activity value of the test set compounds and the mean activity value of the training set compounds. PRESS represents the sum of square deviation between the actual activity and the predicted activity of each compound in the test set.

## Results

### Molecular docking

The x-ray crystal structure of ROCK1 (PDB ID **6E9W**) in complex with a pyridinylbenzamide derivative reported by Hobson *et al*.^31^ was used for the docking study of compound **11**, **17** and **47**. The docking protocol was validated by redocking the co-crystal ligand into the apo-receptor of ROCK1. The re-docked ligand pose showed a root-mean-square deviation (RMSD) value of 1.07 Å.

**Figure 3:**
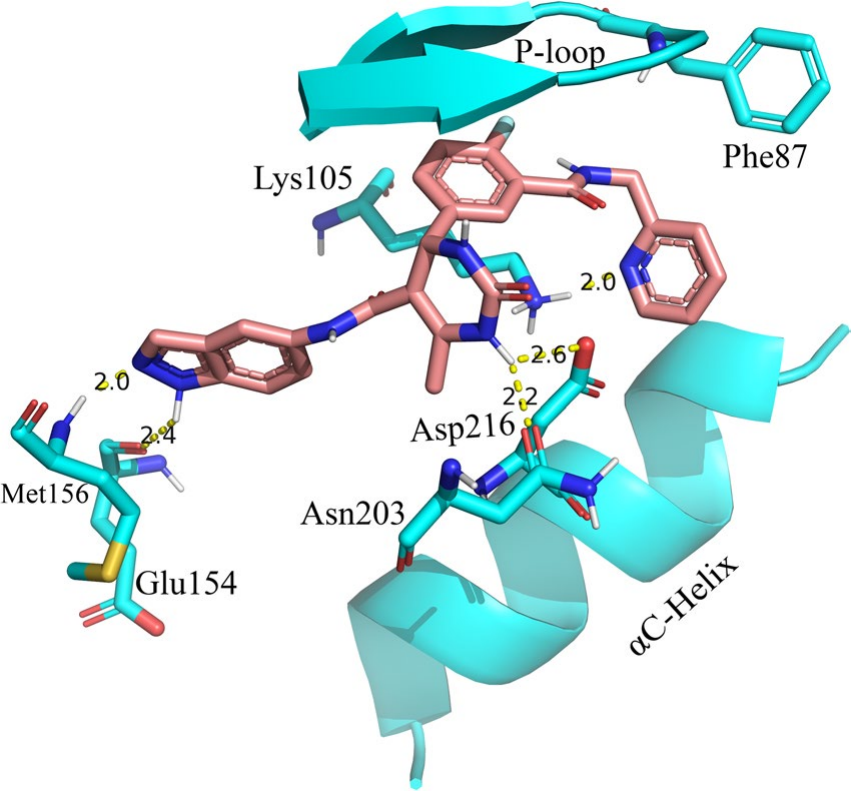
The docked conformation of the most active compound for ROCK1 (compound **11**) inside the active site of ROCK1. H-bond interactions were represented as yellow dotted lines.

Docking of the most active compound for ROCK1 (compound **11**) resulted in 100 conformations. The docking results were analyzed and a pose was selected based on low binding energy and H-bond interactions. The binding site of ROCK1 consisted of residues Gly85, Ala86, Phe87, Lys105, Leu106, Met156, Tyr155, Glu154, Ala215, Asp216, Glu124, Phe120, Phe217, and Leu107. Analysis of the non-bonded interactions showed that the compound **11** formed H-bond interactions with the Glu154 and Met156 at the hinge region, Asn203, and Asp216 at the ribose subsite and Lys105 at the phosphate binding site of ROCK1. The interactions between compound **11** and the binding site residues of ROCK1 are shown in Fig. 3.

Docking studies of compound **17** (most selective compound) and compound **47** (most active compound for GRK2) were also performed to understand the binding modes of the inhibitors inside ROCK1. In the docking of compound **17** with ROCK1, the compound **17** formed H-bond interactions with Met156 at the hinge region and also with Arg84 and Phe87 at the P-loop. Analysis of the docking results for compound **47** with ROCK1 showed that the benzodioxole, piperidine, and pyrazole of compound **47** formed H-bond interactions with the ROCK1 residues Met156, Asp160, and Gly88 respectively. The interactions of the compound **17** and **47** with the binding site residues of ROCK1 are shown in Fig. S4a,b (Supplementary Material) respectively.

From the docking studies, it was observed that compound **11**, **17** and **47** formed H-bond interaction with Met156 at the hinge region of ROCK1. This interaction with the hinge region was considered to be important as it anchors the inhibitor inside the receptor and also induces significant conformational changes in the kinase domain^61^. The docked structures of compound **11**, **17** and **47** inside ROCK1 were used for molecular dynamics simulations studies to understand the dynamic interactions between the inhibitors and ROCK1.

### Molecular dynamics (MD) simulation

**Figure 4:**
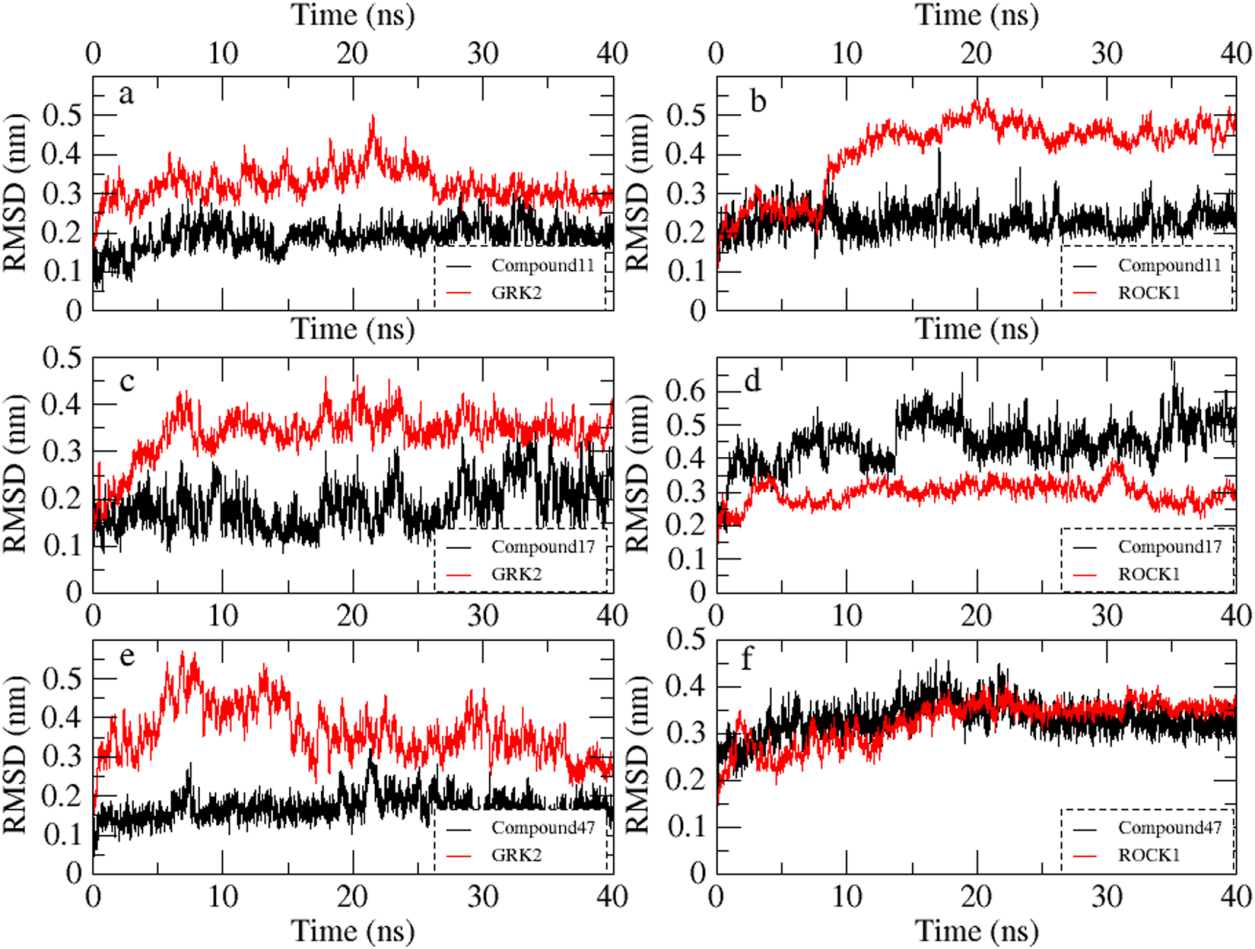
The RMSD diagrams for the 40 ns MD simulation runs. MD production run for each protein-ligand complex was performed once only. (**a**) Compound 11 with GRK2. (**b**) Compound **11** with ROCK1. (**c**) Compound **17** with GRK2. (**d**) Compound **17** with ROCK1. (**e**) Compound **47** with GRK2. (**f**) Compound **47** with ROCK1.

**Figure 5:**
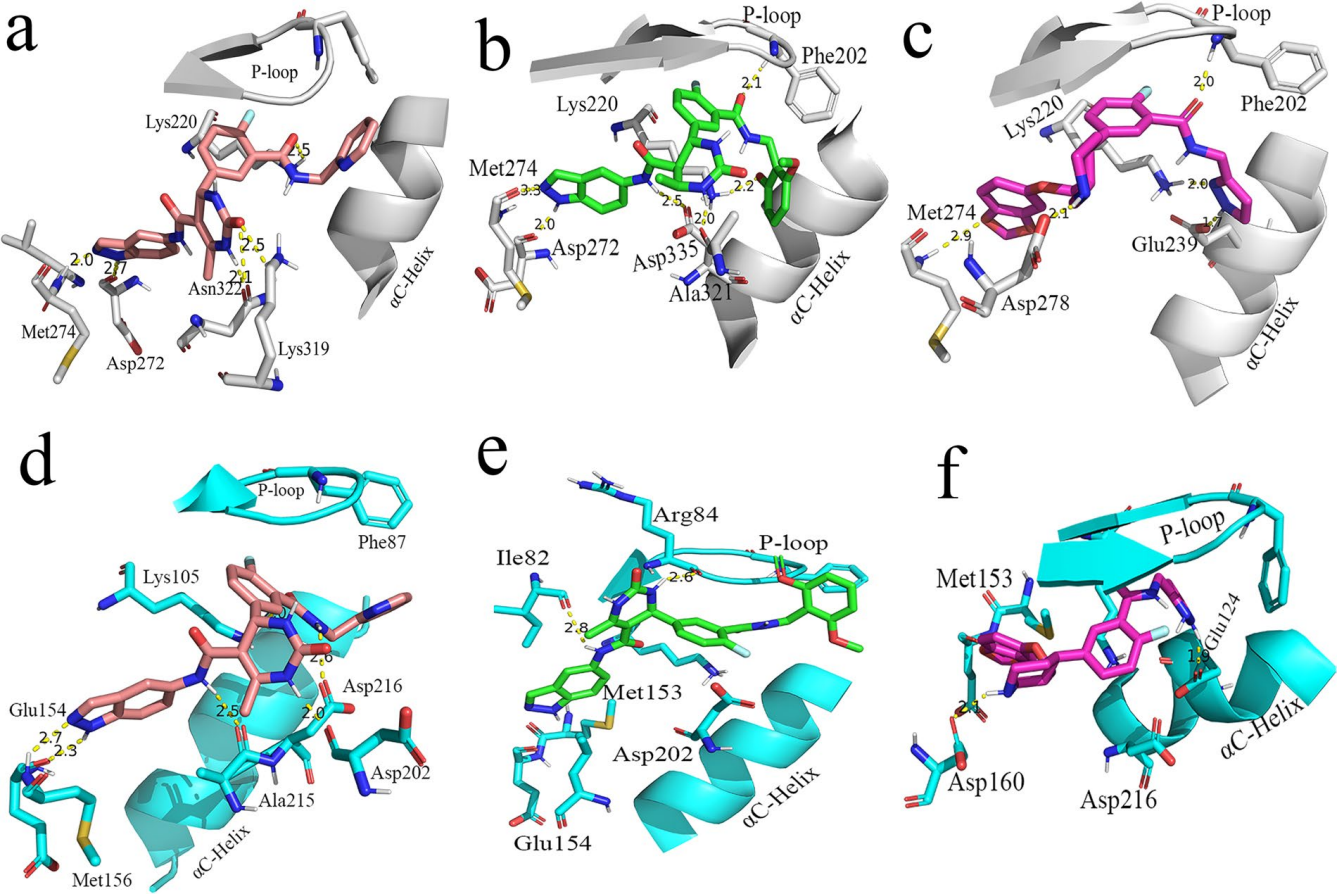
H-bond interactions between the compound **11** (salmon), 17 (green) and **47** (magenta) with GRK2 and ROCK1. Snapshots were collected after 40 ns simulations. The GRK2 and ROCK1 residues are shown in grey and cyan colours respectively. H-bond interactions were represented as yellow dotted lines. (**a**) Compound **11** with GRK2. (**b**) Compound **17** with GRK2. (**c**) Compound **47** with GRK2. (**d**) Compound **11** with ROCK1. (**e**) Compound **17** with ROCK1. (**f**) Compound **47** with ROCK1.

**Figure 6:**
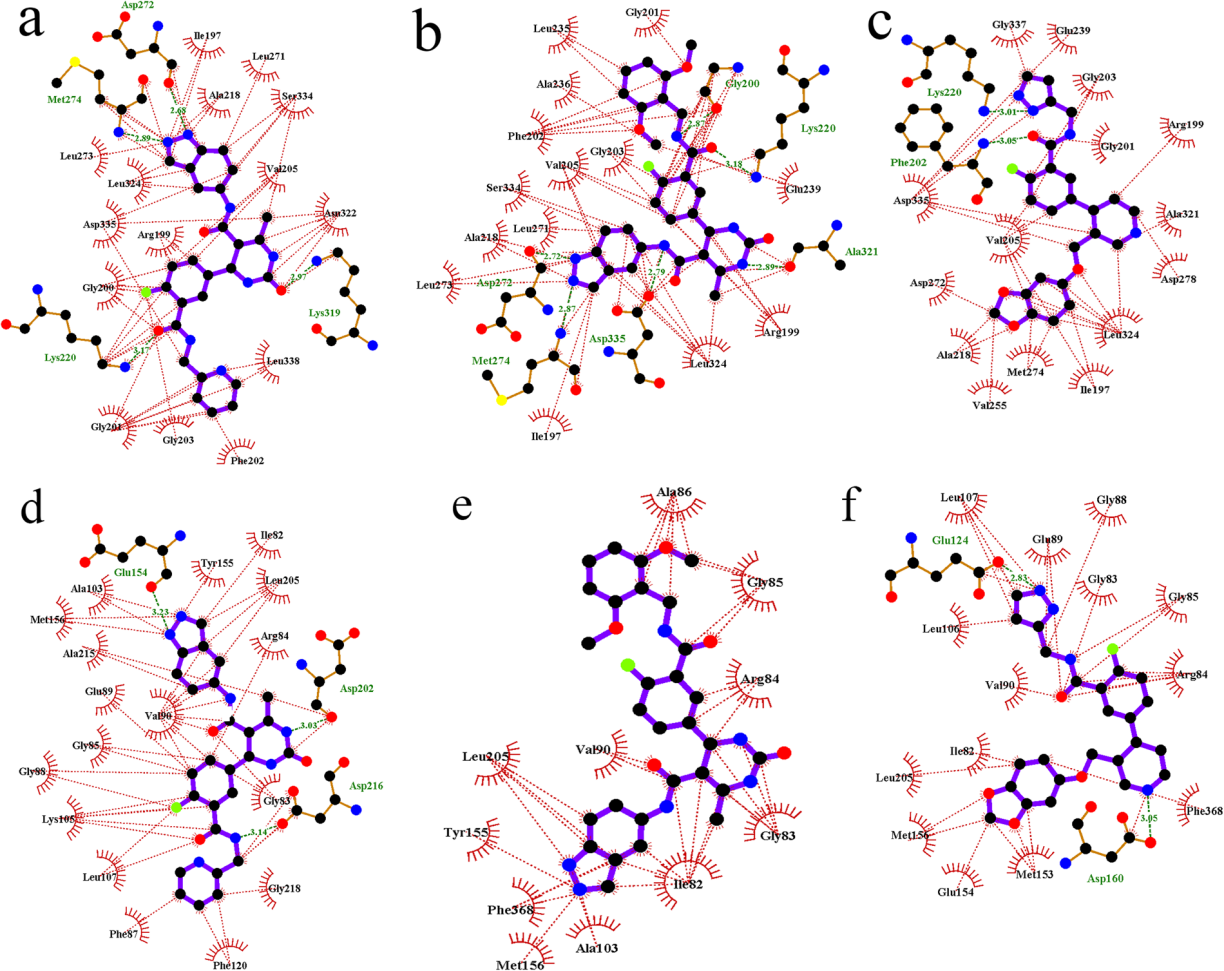
Hydrophobic interactions between compound 11, 17, and 47 with GRK2 and ROCK1. Snapshots were collected after 40 ns simulations. Hydrophobic interactions are represented as red dotted line. H-bond interactions are represented as green dotted lines. (**a**) Compound **11** with GRK2. (**b**) Compound **17** with ROCK1. (**c**) Compound **47** with GRK2. (**d**) Compound **11** with ROCK1. (**e**) Compound **17** with ROCK1. (**f**) Compound **47** with ROCK1.

During the MD simulation studies of compound **11**, **17** and **47** with GRK2, the crystal structure having PDB ID **5HE0** (compound **11**-GRK2 complex), **5HE2** (compound **17**-GRK2 complex) and **5UKM** (compound **47**-GRK2 complex) were used as initial structures^13^. The interactions observed from the crystal structure of compound **11**, **17** and **47** with GRK2 are shown in Fig. S5 (Supplementary Materials). In the MD studies of compound **11**, **17** and **47** with ROCK1, the inhibitor-protein complex structures obtained from the docking studies were used as starting structures. The root-mean-square deviation (RMSD) values of the inhibitors and proteins for the 40 ns MD simulations are shown in Fig. 4. The snapshots of the inhibitor-protein complexes after 40 ns MD simulations were extracted and analyzed to understand the non-bonded interactions between the inhibitors and the receptors. Analysis of the H-bond interactions and hydrophobic interactions were shown in Figs 5 and 6 respectively.

The compound **11** showed H-bond interactions with the GRK2 binding site residues Met274, Asp272, Asn322, Lys 319 and Lys220 as shown in Fig. 5a. The interactions with Met274, Asp272, and Asn322 were observed in the crystal structure of compound **11** with GRK2 (PDB ID **5HE0**). Compound **11** also formed hydrophobic interactions with the GRK2 binding site residues Ile197, Gly200, Gly201, Gly203, Val205, Ala218, Asn322 and Leu324 as shown in Fig. 6a. In the MD study of compound **11** with ROCK1, H-bond interactions between compound **11** and the binding site residues Glu154, Met156, Ala215, Asp202, and Asp216 were observed. Hydrophobic interactions were also observed between compound **11** and the binding site residues Gly83, Gly85, Gly88, Val90, Phe120, and Leu205. The H-bond interactions and hydrophobic interactions between compound **11** and the binding site residues of ROCK1 are shown in Figs 5d and 6d respectively. From the analysis, it was observed that compound **11** formed stable H-bond interactions and hydrophobic interactions with both GRK2 and ROCK1, which could be a possible reason behind the high activity value of the compound for both GRK2 (pIC_50_ = 6.8) and ROCK1 (pIC_50_ = 7.9).

In the MD study of the most selective compound (compound **17**) with GRK2, the compound **17** formed H-bond interactions with the GRK2 binding site residues Met274, Asp272, Asp335, Ala321, Lys220, and Phe202. All these H-bond interactions, except the interaction with Lys220, were also observed in the crystal structure of compound **17** with GRK2 (PDB ID **5HE2**). Compound **17** also formed hydrophobic interactions with residues Arg199, Phe202, Gly203, Val205, Leu235, Leu273 and Leu324 of the GRK2 binding site. The H-bond interactions and hydrophobic interactions of compound **17** with GRK2 are shown in Figs 5b and 6b respectively. In ROCK1, compound **17** formed H-bond interactions with Ile82 and Arg84. The loss of the crucial H-bond interactions with Met156 (hinge region) and Phe87 (P-loop) in ROCK1 indicated that compound **17** was unable to form stable interactions with ROCK1. The H-bond interactions and hydrophobic interactions of compound **17** with ROCK1 are shown in Figs 5e and 6e respectively. It was also observed that the dimethoxybenzene ring of compound **17** extended away from the binding site and was unable to form hydrophobic interactions with residues at hydrophobic subsite of ROCK1. Hydrophobic interactions were observed between compound **17** and residues from the adenine subsite and P-loop of ROCK1 such as Ile82, Gly83, Arg84, Gly85, Ala86, and Leu205.

From the analysis of the MD results for compound **47** with GRK2, it was observed that compound **47** formed H-bond interactions with Met274, Asp278, Glu239, Lys220, and Phe202. In addition to the interactions observed in the co-crystal structure of compound **47** with GRK2 (PDB ID **5UKM**), new interactions with the Met274 (hinge region) and LYS220 (phosphate subsite) were observed. Hydrophobic interactions were also observed between compound **47** and GRK2 binding site residues Ile197, Val205, Gly203, Val205, Met274, Leu324, Asp335, and Gly337. The H-bond interactions and hydrophobic interactions are shown in Figs 5c and 6c respectively. In ROCK1, the compound **47** formed H-bond interactions with residues Met153 and Glu124 however, failed to form interactions with Met156 (Hinge region) and Gly88 (P-loop) of ROCK1. The inability of the compound **47** to form interactions at the hinge region and the P-loop could be the reason why the inhibitor was unable to form stable binding with ROCK1 and extended out of the binding pocket. Compound **47** also formed hydrophobic interactions with residues Gly85, Gly88, Glu89, Val90, Leu106, and Leu107 at the binding site of ROCK1. The H-bond interactions and hydrophobic interactions of compound **47** with ROCK1 are given in Figs 5f and 6f.

The analysis of the binding interactions from the MD studies showed that the most active compound for GRK2 (compound **47**) and the most selective compound (compound **17**) were able to adopt conformations that allow the pyrazole/pyridine rings to form interactions with the Lys220 at the phosphate binding site of GRK2, which was not observed in the interactions with ROCK1. H-bond interaction was also observed between the pyrazole of the compound **47** and Glu239 (αC-Helix) in GRK2 which was not observed in the other inhibitor-protein interactions. The ability of the compound **47** to form stable interactions with Lys220 and Glu239 could be vital for stabilizing the dimethoxybenzene ring at the hydrophobic subsite of GRK2.

### MM/PBSA based free energy calculations

The binding energies of the inhibitor-protein interactions were calculated from the last 5 ns of the 40 ns MD production runs. The results of the binding energy calculations are given in Table 1. The total binding energy for each inhibitor-protein complex was contributed by the following energy terms: van der Waals, electrostatic, polar solvation, and non-polar solvation. From the analyses, we observed that Van der Waals energy and electrostatic energy made the major contribution to the total binding energies. In the interaction of compound **11** with ROCK1, the van der Waals and electrostatic energy values were −225.88 kJ/mol and −120.99 kJ/mol respectively suggesting that van der Waals interactions (hydrophobic interactions) were the major forces in the binding of ROCK1 and its most active compound. In the interaction of compound **47** with GRK2, the contribution of van der Waals energy and electrostatic energy to the total binding energy were −254 kJ/mol and −242.27 kJ/mol respectively suggesting that compound **47** can form favorable van der Waals interactions (hydrophobic interactions) and electrostatic interactions (H-bond interactions) with the binding site residues of GRK2.

**Table 1:** The energy contribution of the various energetic terms (van der Waals energy, electrostatic energy, polar solvation energy, and non-polar solvation energy/SASA) to the total binding energy.

Complexes	Van der Waals (kJ/Mol)	Electrostatics (kJ/Mol)	Polar solvation (kJ/Mol)	SASA (kJ/Mol)	Total Binding Energy (kJ/Mol)
Compound 11- GRK2	−249.62	−85.93	246.63	−24.66	−113.58
Compound 11- ROCK1	−255.88	−120.99	307.92	−24.08	−63.03
Compound 17- GRK2	−262.38	−112.65	275.98	−25.58	−124.63
Compound 17 –ROCK1	−189.84	−76.00	160.00	−20.40	−126.25
Compound 47-GRK2	−254.96	−242.27	293.74	−23.97	−227.48
Compound 47- ROCK1	−225.91	−73.84	190.62	−21.19	−130.34

**Figure 7:**
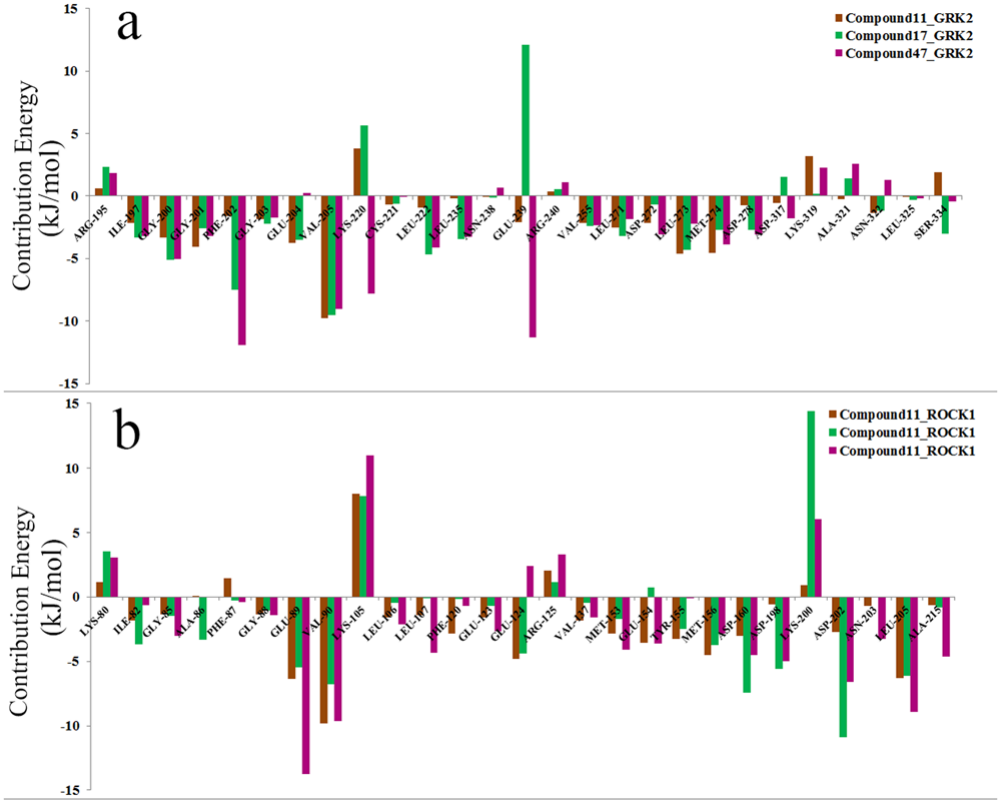
The energy contributions (in kJ/mol) of the key residues to the total binding energy; (**a**) Interaction of compound **11** (brown), **17** (green) and **47** (magenta) with GRK2. (**b**) Interaction of compound **11** (brown), **17** (green) and **47** (magenta) with ROCK1.

The energy contributions of the residues to the total binding energies were calculated for each inhibitor-protein complex to understand the residues that made significant contributions in the inhibitor-protein interactions. The binding energy values for the residues at the binding site of GRK2 that made significant contributions to the total binding energy are shown in Fig. 7a and the energy values for the corresponding residues in ROCK1 are shown in Fig. 7b. In binding of compound **11**, **17** and **47** with GRK2, the residues Gly200, Gly201, Phe202, Val 204 and Lys205 from the P-loop, the residues Leu222 and Glu235 from the phosphate subsite and the residues Leu271, Asp272, Leu273, met274, Asn275 and Asp278 from the adenine subsite made vital contributions to the total binding energies. Whereas in ROCK1, the residues Glu89 and Val90 from the P-loop, Met153, Glu154, Tyr155 and Met156 from the adenine subsite and residues Asp160, Asp 202, Leu205 and Asp216 from the ribose subsite made vital contributions to the total binding energies.

### 3D-QSAR

The CoMFA models for GRK2 and ROCK1 were developed using Sybyl-X 2.1. During the development of the CoMFA model for GRK2, the structure of the most active compound for GRK2 (compound **47**) given in the co-crystallized structure (PDB ID **5UKM**) was used as the template for aligning the dataset compounds. The model was built based on a training set of 30 compounds and the remaining 23 compounds were used for testing the model. The aligned compounds are shown in Fig. 2a. The CoMFA model showed a cross-validated correlation coefficient (*q*^2^) value of 0.67 and non-cross-validated correlation coefficient (*r*^2^) value of 0.92. The statistical results of the CoMFA model are shown in Table 2. The derived CoMFA model showed an LFO value of 0.54 and also showed reasonable BS-*r*^2^ and BS-SD value of 0.96 and 0.03 respectively. During the external validation, the CoMFA model exhibited acceptable predictive ability showing an *r*^2^_pred_ value of 0.61.

**Table 2:** Statistical results of the CoMFA models for GRK2 and ROCK1.

Parameters	CoMFA (GRK2)	CoMFA (ROCK1)
*q* ^2^	0.67	0.59
ONC	5	6
SEP	0.50	0.57
*r* ^2^	0.92	0.94
SEE	0.22	0.2
F value	52.46	35.93
LFO	0.54	0.62
BS *r*^2^	0.96	0.98
BS SD	0.03	0.01
*r* ^2^_*pred*_	0.61	NA
**Influence of different fields** **(%)**		
S	0.49	0.73
E	0.50	0.27

*q*^2^: cross-validated correlation coefficient; ONC: Optimal number of components; SEP: Standard Error of Prediction; *r*^2^: non-cross-validated correlation coefficient; SEE: Standard Error of Estimation; F value: F-test value; *r*^2^; LFO: Leave five out; BS-*r*^2^: Bootstrapping *r*^2^ mean; BS-SD: Bootstrapping Standard deviation; *r*^2^_*pred*_: predictive correlation coefficient; S: Steric; E: Electrostatic; ND: Not Determined.

During the development of the CoMFA model for ROCK1, the average structure of the most active compound for ROCK1 (compound **11**) extracted from the last 5 ns of the 40 ns MD simulation (compound **11**-ROCK1 complex) was used as the template for aligning the dataset compounds. The CoMFA model was build using 21 compounds. The aligned compounds for the CoMFA model for ROCK1 are shown in Fig. 2b. The derived model showed a *q*^2^ value of 0.59 and *r*^2^ value of 0.94. During the model validation, the CoMFA model showed an LFO value of 0.62 and showed reasonable BS-*r*^2^ and BS-SD value of 0.98 and 0.01 respectively. The statistical results are shown in Table 2.

The statistical results from the CoMFA models for GRK2 and ROCK1 suggested that the models have acceptable robustness and predictive ability. The comparison of the actual activity values and the predicted activity values for the CoMFA models for GRK2 and ROCK1 are shown in Tables S2 and S3 (Supplementary Material) respectively. The scatter plot for the CoMFA models are given in Fig. S6 (Supplementary Material).

### Contour map analysis

**Figure 8:**
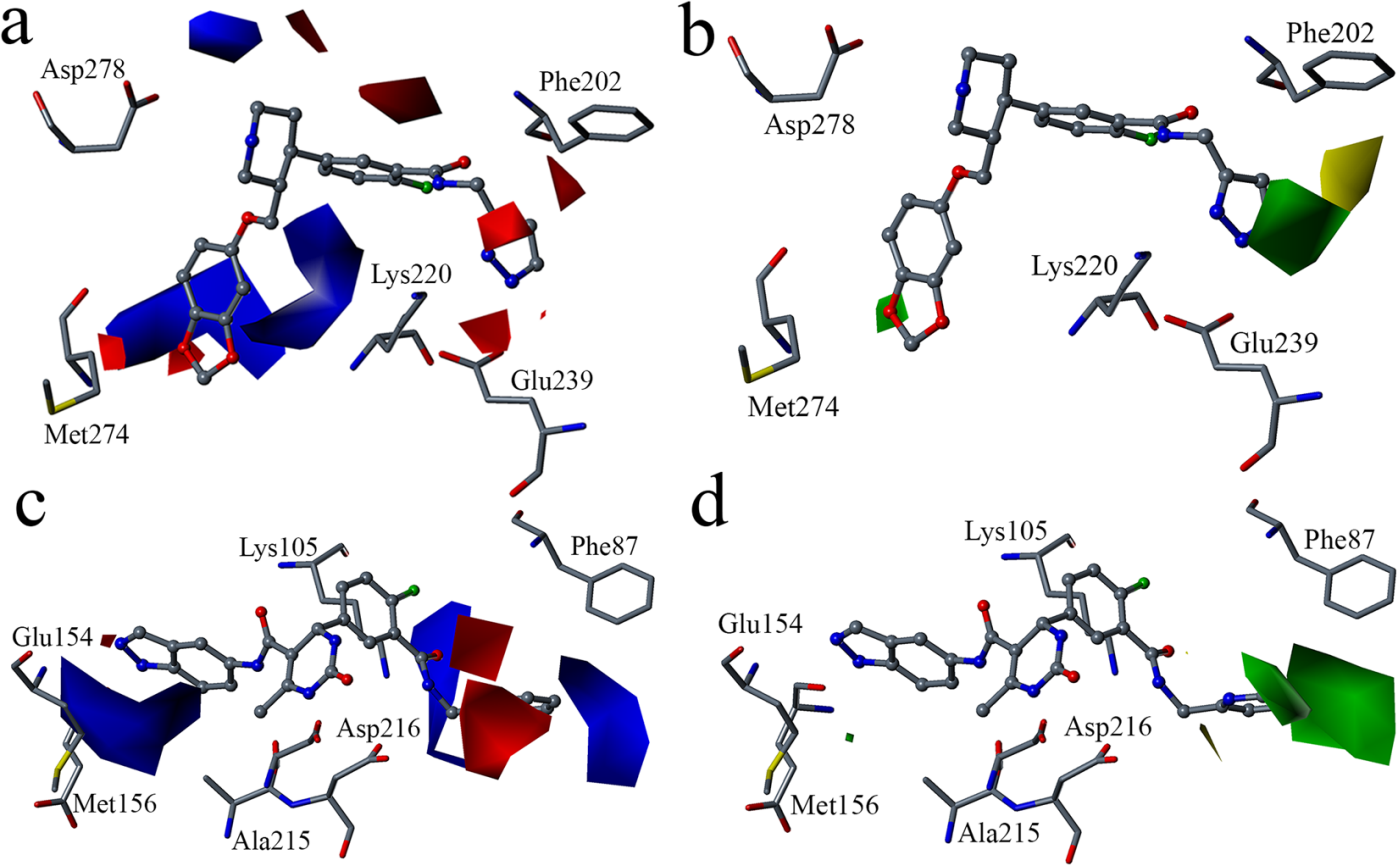
Standard coefficient contour maps obtained from GRK2 and ROCK1 CoMFA analyses. In the electrostatic contour maps, Blue contour indicates electropositive substituent favorable regions and red contour indicates electronegative substituent favorable regions. In the steric contour maps, green contour indicates steric bulk favorable regions and yellow contour indicates steric bulk unfavorable regions. (**a**) Electrostatic contour map for GRK2 CoMFA model with the template compound (compound **47**) as reference. (**b**) Steric contour map for GRK2 CoMFA model with the template compound (compound **47**) as reference. (**c**) Electrostatic contour map for ROCK1 CoMFA model with the template compound (compound **11**) as reference. (**d**) Steric contour map for ROCK1 CoMFA model with the template compound (compound **11**) as reference.

The electrostatic and steric contour maps developed from the CoMFA models for GRK2 and ROCK1 are shown in Fig. 8. In the electrostatics contour maps, the regions favorable to electropositive substituents were shown in blue color contours and the electronegative substituents favorable regions were shown in red color contours. In the steric contour maps, the bulky substituents and non-bulky substituents favorable regions were represented in green and yellow contours respectively.

In the CoMFA contour maps for GRK2, compound **47** (most active compound for GRK2) was used as a reference (Fig. 8a,b). The blue contours observed near the benzodioxole, the piperidine ring and near the linker between the benzodioxole and the piperidine ring suggested that electropositive substituents at these positions are favored. Red color contours were observed near the pyrazole ring, the benzodioxole and near the linker between the benzene ring and the pyrazole ring suggesting that electronegative substituents are favored in these regions. The electronegative and electropositive substituents near the benzodioxole can lead to H-bond interactions with the GRK2 hinge region residues such as Met274 and Asp272 as observed in compound **11**, **17** and **47**. Electronegative substituents at the pyrazole ring can lead to H-bond interaction with Lys220 as observed in compound **17** and **47**. In the steric contour map (Fig. 8b), green contours were observed near the benzodioxole and the pyrazole ring suggesting that bulky substituents are favored in these regions. As the benzodioxole and the pyrazole ring occupied the adenine subsite and the hydrophobic subsite respectively, the presence of bulky substituents may result in favorable non-bonded interactions with residues surrounding the hydrophobic subsite. Yellow contour at the back of the pyrazole ring suggested that extended bulky substituents are not favorable in this region. Extended bulky substituents in this region can cause steric clash with binding site residues. This is exemplified by compound **22**, **30**, **40**, **41** and **42** all of which have relatively lower activity value for GRK2 in the series.

In the CoMFA contour maps for ROCK1, the compound **11** (most active compound for ROCK1) was used as a reference (Fig. 8c,d). The blue contours near the indazole and near the linker between the benzene ring and the pyridine ring in the electrostatic contour map (Fig. 8c) suggested that electropositive substituents at these positions are favorable and may increase the activity of the compounds for ROCK1. Electropositive substituents at the linker between the benzene ring and the pyridine can lead to H-bond interactions with Ala215 as observed in the interaction of compound **11** with ROCK1 (Fig. 5d). The red contour near the pyridine ring suggested that electronegative substituents are favored in that region. Having electronegative substituents at the pyridine ring can lead to favorable H-bond interactions with Lys105 as observed in the interaction compound **11** with ROCK1. Green contour was observed near the pyridine ring, suggesting that bulky substituents are favorable in that region (Fig. 8d).

From the analysis of the CoMFA contour maps for GRK2 and ROCK1, it was observed that electronegative substituents near the benzodioxole and near the piperidine ring and having bulky substituents near the piperidine ring increased the activity for both GRK2 and ROCK1. Whereas, having electropositive substituents at the piperidine ring, having electropositive and electronegative substituents at the benzodioxole and having electronegative substituents near the amide linker between the benzene ring and the pyrazole ring are favorable for increasing the activity for GRK2 with selectivity over ROCK1.

## Discussions

From the analysis of the inhibitor-protein interactions from the MD simulation results, it was observed that compound **11**, **17** and **47** were able to form stable H-bond interactions with residues from the hinge region (Met274 and Asp272) and the ribose subsite (Asp278, Lys319, Asn322, and Asp335) of GRK2 which anchored the inhibitors at the binding site (Fig. 5a–c). Compound **11** also formed H-bond interactions with residues from the hinge region (Glu154) and the ribose subsite (Asp216 and Asp202) of ROCK1 (Fig. 5d). However, compound **17** and **47** could not form stable interactions with residues at the hinge region and at the ribose subsite of ROCK1 (Fig. 5e,f), which could be a reason behind the poor activity of these compounds towards ROCK1.

**Figure 9:**
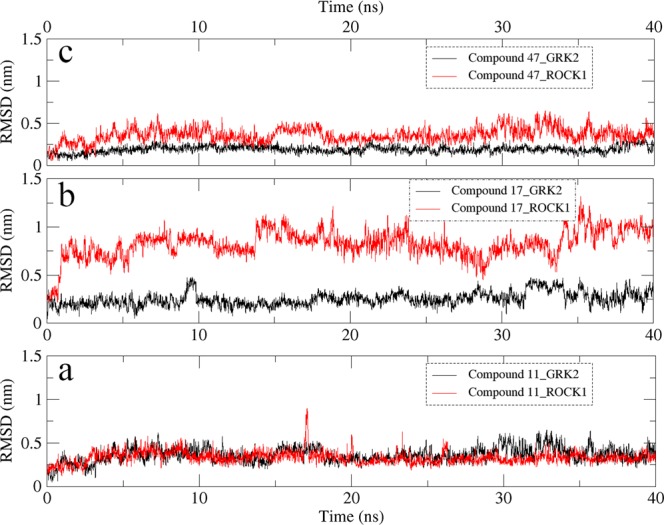
RMSD values of pyridine ring (compound **11**), dimethoxybenzene ring (compound **17**) and pyrazole ring (compound **47**) inside GRK2 and ROCK1 for 40 ns MD simulations. MD production run for each protein-ligand complex was performed once only. (**a**) RMSD of pyridine ring of compound **11** at the hydrophobic pocket of GRK2 (black) and ROCK1 (red). (**b**) RMSD of dimethoxybenzene ring of compound **17** at the hydrophobic pocket of GRK2 (black) and ROCK1 (red). (**c**) RMSD of pyrazole ring of compound **47** at the hydrophobic pocket of GRK2 (black) and ROCK1 (red).

In both compound **17** and compound **47**, the oxygen at the amide linker between the benzene ring and the dimethoxybenzene ring/pyrazole ring formed H-bond interactions with the nitrogen of Phe202 (P-loop) and extended the dimethoxybenzene/pyrazole ring into the hydrophobic pocket of GRK2 (Fig. 5b,c). This allowed the dimethoxybenzene ring/pyrazole rings to form H-bond interactions with Lys220 (phosphate subsite) of GRK2. These H-bond interactions with the P-loop (Phe87) and the phosphate subsite (LYS105) were not observed in ROCK1. These interactions with the Phe202 and Lys220 could be crucial for the binding stability of compound **17** and compound **47** inside GRK2. To investigate the influence of the interactions with the Phe202 and Lys220 on the stability of the dimethoxybenzene/pyrazole ring inside the hydrophobic pockets, we calculated the RMSD of the pyridine ring (compound **11**), dimethoxybenzene ring (compound **17**) and pyrazole ring (compound **47**) inside the hydrophobic subsites of GRK2 and ROCK1. MD production run for each protein-ligand complex was performed once only. The outcomes of the RMSD calculations for each protein-ligand complex are shown in Fig. 9. The pyridine ring of Compound **11** showed an average RMSD value of 0.36 Å and 0.33 Å inside GRK2 and ROCK1 respectively (Fig. 9a). Though compound **11** formed multiple H-bond interactions with the binding site residues of GRK2 and ROCK1, it did not form interactions with Lys220 (GRK2) or Lys105 (ROCK1) which could be the reason behind the increased in the average RMSD values of 0.36 Å and 0.33 Å for GRK2 and ROCK1 respectively. The pyrazole of compound **47** showed an average RMSD value of 0.19 Å and 0.37 Å in GRK2 and ROCK1 respectively (Fig. 9c). The low average RMSD value of the pyrazole ring inside GRK2 suggested that the pyrazole of compound **47** was stably locked at the hydrophobic subsite. This stability may be attributed to the H-bond interactions with Lys220 and Glu239 at the hydrophobic subsite of GRK2. The biggest difference in the RMSD value was observed for the dimethoxybenzene ring of compound **17** which showed an average RMSD value of 0.25 Å and 0.85 Å inside GRK2 and ROCK1 respectively (Fig. 9b). The difference in the average RMSD values may be attributed to the fact that compound **17** was able to form interactions with Phe202 and Lys220 in GRK2 which stabilized the dimethoxybenzene ring inside the hydrophobic subsite, however, in ROCK1, the dimethoxybenzene ring extended out of the hydrophobic subsite and did not form interactions at the hydrophobic subsite.

The H-bond interactions with Phe202 and Lys220 could be the reason behind the stability of compound **17** and **47** in GRK2, resulting in higher activity of the compounds for GRK2. These observations suggested that the H-bond formation with the Phe202 and Lys220 may be crucial for the stability of the inhibitors at the hydrophobic pocket of GRK2 and could potentially lead to selective inhibition of GRK2 over ROCK1.

## Conclusions

In this study, we have used molecular docking, molecular dynamics simulation, free energy calculation and 3D-QSAR methods to study a series of 53 paroxetine-like inhibitors to understand the structural properties that drive the inhibitory preference for GRK2 over ROCK1. The observations from the MD studies suggested that H-bond interactions of the inhibitors with the residues at hinge regions and ribose subsites are crucial for anchoring the inhibitors at the binding site in GRK2 and ROCK1. It was also observed that H-bond interactions with Phe202 and Lys220 increased the stability of the inhibitors at the hydrophobic subsite of GRK2. Hence, H-bond interactions with Phe202 and Lys220 were considered to be vital for the selective inhibition of GRK2. Free energy calculations of the inhibitor-protein interactions suggested that van der Waals and electrostatic energies were the major contributors to the total binding energies in GRK2 and ROCK1. Residue-wise energy decompositions indicated that van der Waals interactions and electrostatic interactions with residues Phe202, Val205, Lys220, and Glu239 were important for the inhibition of GRK2 with selectivity over ROCK1. Analysis of the contour maps from the 3D-QSAR models suggested that having electropositive substituents at the piperidine ring, electronegative and electropositive substituents at the benzodioxole and electronegative substituent near the amide linker between the benzene ring and the pyrazole ring were favorable in GRK2 and may lead to increased inhibitor activity for GRK2 with selectivity over ROCK1.

The statistical results and scientific observations reported in this study contributed in understanding the structural properties required for the selective inhibition of GRK2 with selectivity over ROCK1. The outcome of this study could be useful in designing potent GRK2 inhibitors with selectivity over ROCK1 for therapeutic intervention of heart failure diseases.

## Supplementary information

Supplementary material

## Data Availability

All relevant data are contained within the manuscript and the supplementary material. Additional raw data will be available upon request.
