# Supplementary material for: Computational study of paroxetine-like inhibitors reveals new molecular insight to inhibit GRK2 with selectivity over ROCK1
Source: Sci Rep. 2019 Sep 10;9:13053. doi: 10.1038/s41598-019-48949-w (PMC6736929; doi:10.1038/s41598-019-48949-w)

Computational study of paroxetine-like inhibitors reveals new molecular insight to inhibit GRK2 with selectivity over ROCK1

Seketoulie Keretsu[a], Swapnil P. Bhujbal[a], Seung Joo Cho[a,b,*]

[a] *Department of Biomedical Sciences, College of Medicine, Chosun University, Gwangju 501-759, Republic of Korea*

[b] *Department of Cellular·Molecular Medicine, College of Medicine, Chosun University, Gwangju 501-759, Republic of Korea*

[*] Author for correspondence

Address: College of Medicine, Chosun University, 375 Seosuk-dong, Dong-gu Gwangju 501-759, Republic of Korea;

E-mail:chosj@chosun.ac.kr;

Telephone: +82-62-230-7482 (office) +82-11-479-1010 (cell phone

Supplementary Materials

Table S1. Comparison of the residues at the adenine subsite, polyphosphate subsite, ribose subsite and hydrophobic subsite for GRK2, ROCK1 and ROCK2

Subsites		GRK2	ROCK1	ROCK2
**Adenine Subsite**		Met274, Asn275, Gly276, Gly277, Asp278, Leu279, His280	Met156, Pro157, Gly158, Gly159, Asp160, Leu161, Val162	Met172, Pro173, Gly174, Gly175, Asp176, Leu177, Val178.
**Polyphosphate Subsite**		Tyr217, Ala218, Met219, Lys220, Cys221, Leu222	Tyr102, Ala103, Met104,Lys105,Leu106, Leu107	Tyr118, Ala119, Met120, Lys121, Leu122, Leu123
**Ribose Subsite**		Gly276, Gly277, Asp278, Leu279, His280, Tyr281, His282, Leu283, Ser284	Gly156, Gly159, Asp160, Leu161, Val162, Asn163, Leu164, Met165	Gly174, Gly175, Asp176, Leu177, Val178, Asn179, Leu180, Met181,
**Hydrophobic Subsite**	**P-loop**	Ile197, Gly198, Arg199, Gly200, Gly201, Phe202, Gly203, Glu204, Val205	Ile98, Gly99, Arg100, Gly101, Ala102, Phe103, Gly104, Gly105, Val106	Ile82, Gly83, Arg84, Gly85, Ala86, Phe87, Gly88, Glu89, Val90
	**αC-Helix**	Thr234, Leu235, Ala236, Leu237, Asn238, Glu239, Arg240, Ile241, Met242, Leu243, Ser244, Leu245, Val246, Ser247	Ser118, Ala119, Phe120, Phe121, Trp122, Glu123, Glu124, Arg125, Asp126, Ile127, Met128, Ala129, Phe130, Ala131	Ser134, Ala135, Phe136, Phe137, Trp138, Glu139, Glu140, Arg141, Asp142, Ile143, Met144, Ala145, Phe146, Ala147
	**DFG/DLG motif**	Asp335, Leu336, Gly337	Asp216, Phe217, Gly218	Asp232, Phe233, Gly234

Table S2. Experimental and predicted pIC_{50} values with their residuals of CoMFA for GRK2

Compound	Actual pIC_{50}	GRK2 CoMFA	
		Predicted pIC_{50}	Residual
1*	5.9	6.0	-0.1
2*	6.1	5.8	0.3
3	4.7	4.9	-0.2
4	5.4	5.4	-0.1
5*	6.2	6.8	-0.7

6	6.7	6.8	-0.1
7	6.7	6.6	0.1
8	7.3	7.2	0.0
9*	6.4	6.1	0.3
10	6.4	6.6	-0.3
11	6.8	6.8	0.0
12	6.6	6.5	0.0
13	5.3	5.2	0.1
14*	6.6	6.2	0.4
15	6.9	7.1	-0.2
16*	7.2	7.5	-0.4
17*	6.9	7.1	-0.2
18	5.9	6.0	0.0
19	5.6	5.4	0.2
20	5.7	5.7	0.0
21*	6.6	6.7	-0.1
22	4.6	4.5	0.1
24*	6.3	5.6	0.8
25	6.2	6.4	-0.2
26	5.7	5.5	0.2
27*	6.1	6.1	0.0
28	5.6	5.6	-0.1
29	5.8	6.0	-0.2
30*	5.7	5.9	-0.2
31*	4.9	4.9	0.0
32	5.7	5.9	-0.3

33	5.7	5.8	0.0
34	5.7	5.7	0.0
35*	4.4	5.1	-0.7
36	5.6	5.6	0.0
37*	6.2	6.6	-0.4
38	5.8	5.9	-0.1
39	5.7	5.7	0.0
40*	5.5	5.9	-0.4
41*	5.5	5.7	-0.2
42	5.5	5.6	-0.1
43	5.2	5.2	0.1
44*	5.2	5.9	-0.7
45	6.1	6.1	0.0
46	6.2	6.2	0.0
47	7.5	7.0	0.6
48	6.1	6.7	-0.6
49	7.5	7.1	0.4
50*	5.9	5.7	0.2
51	5.7	5.4	0.2
52	6.4	6.4	0.0
53*	4.8	5.7	-0.9

* Test set compounds

Table S3. Experimental and predicted pIC_{50} values with their residuals of CoMFA for ROCK1.

**Compound**	**Actual pIC_{50}**	**ROCK1 CoMFA**	
		**Predicted pIC_{50}**	**Residual**
2	7.0	7.1	-0.1
3	6.7	6.6	0.1
4	6.3	6.3	0.0
5	7.2	7.1	0.0
6	7.7	7.4	0.3
7	7.0	6.6	0.3
8	7.2	7.3	-0.1
9	7.0	7.0	0.0
10	7.3	7.3	0.0
11	8.0	8.2	-0.3
12	7.6	7.6	0.0
13	7.1	7.1	0.0
14	7.9	7.9	0.0
15	5.2	5.7	-0.5
16	5.2	5.1	0.2
19	5.7	5.2	0.5
20	6.8	6.9	-0.1
21	6.5	6.5	-0.1
22	6.3	6.4	-0.1
23	6.4	6.4	-0.1
24	6.7	6.7	-0.1

Figure S1. The catalytic domain (grey) of GRK2 with the compound **47** (magenta) binded at its active site (PDB ID **5UKM**). The adenine subsite, ribose subsite, polyphosphate subsite and hydrophobic subsite are indicated with black rectangular boxes. The DFG/DLG motif and the Lys220 which are conserved in most AGC kinases are shown in stick representation (deepblue).

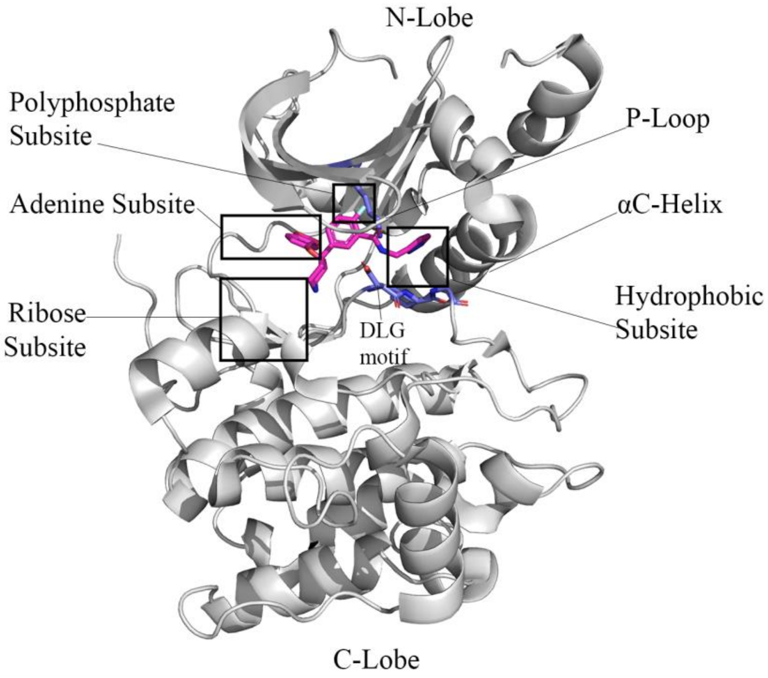

Figure S2. The alignment of the amino acid sequences in the kinase domains of GRK2 and ROCK1. Identical and positive matches in the sequences are highlighted by red and green colours respectively.

Protein	Residue number	**Identities:** 89/271 (33 %) **Positives:** 143/271 (52%) — **Sequences**	Residue number
**GRK2**	191	FSVHR I IGRGGFGEVYGCRKADTGKMYAMKCLDKKRIKMKQGETLALNERIMLSLVSTGD	250
**ROCK1**	76	YEVVKVIGRGAFGEVQLVRHKSTRK V YAMKLLSKFEMI KRSDSAF FWEERDIMAFANS- -	133
**GRK2**	251	CPF I VCMSYAFHTPDKLS FI L DLMNGGDLHYHLSQHGVFSEADMRFYAAEI I LGLEHMHN	310
**ROCK1**	134	- PW VVQLFYAFQDDRYLYMVMEYMPGGDLVNLMSNYDV-PEKWARFYTAEVVLALDA IHS	191
**GRK2**	311	RFVVYRDLKPAN ILLDEHGHVR I SDLGLACDF S KK - - KPHAS VGTHGYMAPEVLQKGVA	367
**ROCK1**	192	MGFIHRDVKPDNMLLDKSGHLKLADFGTCMKMNKEGMVRCDTAVGTPDY I SPEVLKSQGG	251
**GRK2**	368	- - - YDSS ADWF SLGCMLFKLL RGHSPFRQHKTKDKHEIDRMTLTMAVELPD- -SFSPELR	422
**ROCK1**	252	DGYYGRECDWWSVGVFLYEMLVGDTPFYADS LVGTYS–KIMNHKNS LTFPDDNDISKEAK	310
**GRK2**	423	SLLEGLLQRDVNRRLGCLGRGAQEVKES PFF	453
**ROCK1**	311	NLI CAFL T- DREVRLG - -RNGVEE I KRHLFF	338

Figure S3. The common substructure used in aligning the dataset compounds during the development of the CoMFA Models for GRK2 and ROCK1.

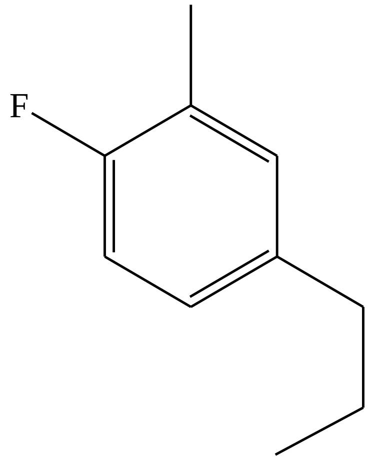

Figure S4. The docked conformations of the most selective compound (compound **17**) and the most active compound for GRK2 (compound **47**) inside the active site of ROCK1. H-bond interactions were represented as yellow dotted lines. **(a)** Compound **17** with ROCK1 **(b)** Compound **47** with ROCK1.

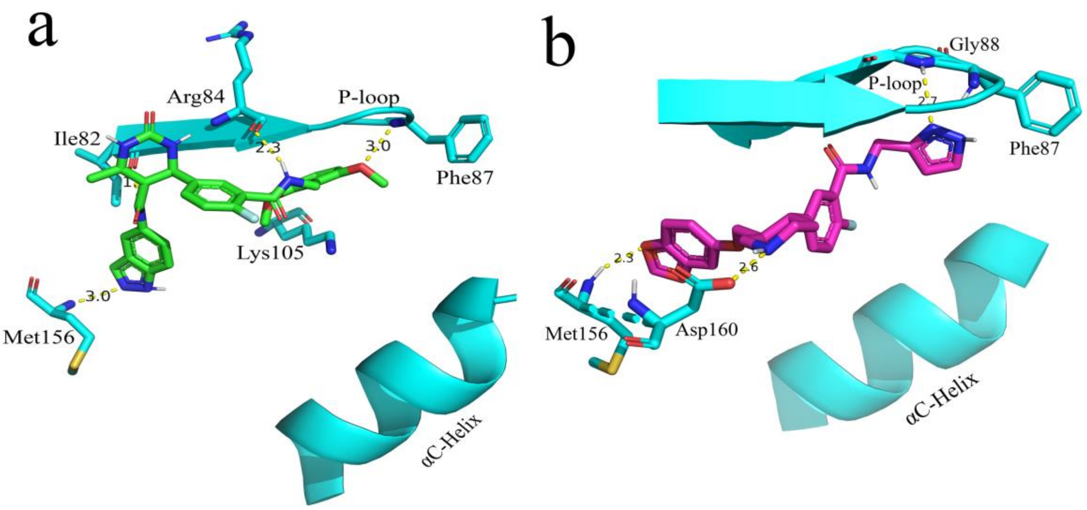

Figure S5. The interactions observed in the crystal structures of compound **11**, **17** and **47** with GRK2. H-bond interactions were represented as yellow dotted lines. **(a)** Compound **11** and GRK2 (PDB ID **5HE0**) **(b)** compound **17** and GRK2 (PDB ID **5HE2**) **(c)** compound **47** and GRK2 (PDB ID **5UKM**)

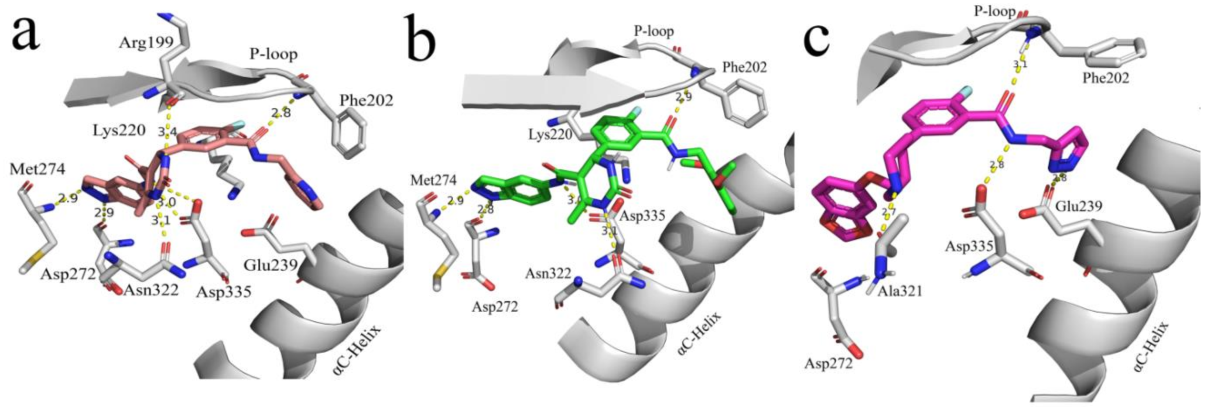

Figure S6. Scatterplot generated from the CoMFA models. (**a**) Scatterplot of the CoMFA model for GRK2. (**b**) Scatterplot of the CoMFA model for ROCK1. The values on x-axis and y-axis represent the predicted pIC_{50} value and the actual pIC_{50} values respectively.

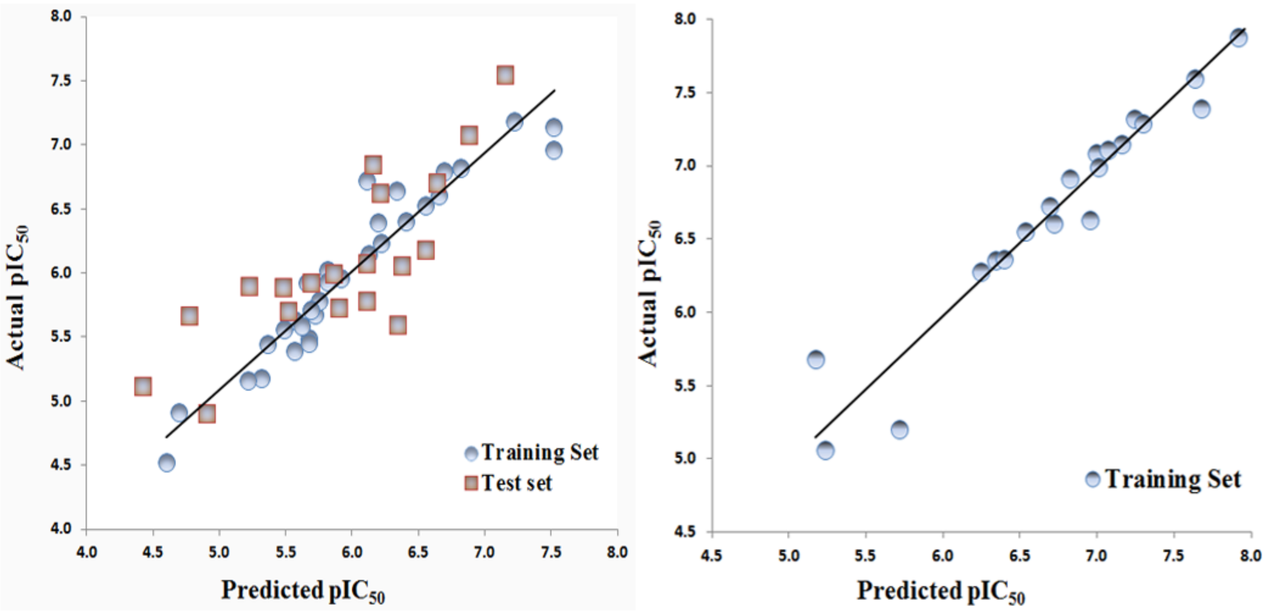

Supplement: Supplementary file 1 — Supplementary material [file 41598_2019_48949_MOESM1_ESM.pdf]
